# 8-Iso-Prostaglandin F2α and Lipoxygenase Gene Expression as Candidate Molecular Markers of Training Adaptation in Professional Volleyball Players: A Pilot Study

**DOI:** 10.3390/ijms27167104

**Published:** 2026-08-08

**Authors:** Krystian Baran, Rafał Podgórski, Michalina Grzesik-Pietrasiewicz, Wojciech Czarny, Paweł Król, Julia Połeć, Élvio Rúbio Gouveia, Marek Cieśla

**Affiliations:** 1 Faculty of Medicine, University of Rzeszów, 35-959 Rzeszów, Poland; rpodgorski@ur.edu.pl (R.P.); migrzesik@ur.edu.pl (M.G.-P.); mciesla@ur.edu.pl (M.C.); 2 Faculty of Physical Culture Studies, University of Rzeszów, 35-959 Rzeszów, Poland; wczarny@ur.edu.pl (W.C.); pkrol@ur.edu.pl (P.K.); 3 Student Science Club “Molecule”, Faculty of Medicine, University of Rzeszów, 35-959 Rzeszów, Poland; jp131108@stud.ur.edu.pl; 4 Department of Physical Education and Sport, University of Madeira, 9020-105 Funchal, Portugal; erubiog@staff.uma.pt

**Keywords:** molecular markers, redox signaling, lipid peroxidation, 8-iso-PGF2α, lipoxygenases, volleyball, training adaptation

## Abstract

Intensive training induces redox and inflammatory signaling that may facilitate adaptation but can also reflect excessive physiological strain. In this exploratory pilot longitudinal study, we assessed whether 8-iso-prostaglandin F2α (8-iso-PGF2α), a lipid peroxidation marker, and transcripts related to arachidonic acid lipoxygenase pathways change during a 10-week preparatory training period in elite female volleyball players. Twelve professional athletes were sampled at baseline after a training break and after training completion. 8-iso-PGF2α was quantified by gas chromatography–mass spectrometry (GC-MS), and the relative expression of arachidonate 5-lipoxygenase (*ALOX5*), arachidonate 12-lipoxygenase (*ALOX12*), and arachidonate 15-lipoxygenase (*ALOX15*) genes was measured by reverse transcription quantitative polymerase chain reaction (RT-qPCR) in leukocytes and plasma-derived RNA. Training increased 8-iso-PGF2α from 0.06 [0.05–0.09] to 0.16 [0.08–0.22] ng/mL (*p* = 0.03). *ALOX5* expression increased in leukocytes (0.80 ± 0.29 vs. 1.00 ± 0.29; *p* = 0.02) and plasma-derived RNA (0.03 [0.02–0.06] vs. 0.06 [0.05–0.11]; *p* = 0.04), whereas no statistically detectable pre-to-post changes were observed for *ALOX12* and *ALOX15* expression. Given the exploratory design and small sample size, these results should be interpreted as hypothesis-generating. The observed changes suggest that 8-iso-PGF2α and *ALOX5* are associated with physiological responses to preparatory training and exercise-induced lipid oxidative remodeling; however, their potential utility for monitoring training adaptation requires validation in larger, prospectively designed controlled cohorts.

## 1. Introduction

Studies conducted in athletes play a crucial role in contemporary research on health and exercise physiology, as they enable the assessment of the body’s adaptation to increase metabolic demands and the identification of mechanisms underlying physical performance and recovery. Objective evaluation of training-induced changes is made possible through the analysis of biochemical biomarkers. Markers of oxidative stress, inflammation, and cellular damage provide insight into the balance between adaptive processes and the potential detrimental effects of intense physical exertion [1,2,3]. The levels of these indicators are influenced by multiple factors, including training status, exercise intensity and duration, dietary habits, and genetic predisposition [4,5]. Moreover, studies on epigenetic markers in athletes indicate that physical activity can modulate DNA methylation and gene expression related to metabolism and inflammatory responses, suggesting the involvement of epigenetic mechanisms in training adaptation [6,7,8].

An imbalance between the production of reactive oxygen species (ROS) and the body’s ability to neutralize them through antioxidant defense systems, referred to as oxidative stress, can lead to damage of cellular structures, including lipids, proteins, and DNA [9,10]. Intense physical exercise increases ROS production, which may transiently exacerbate oxidative stress; however, regular training promotes adaptive responses, including the upregulation of endogenous antioxidant systems [11]. Regular training should not be interpreted solely as a source of oxidative damage. Current models of exercise biology emphasize that reactive oxygen and nitrogen species generated during muscle contraction also act as signaling molecules that regulate mitochondrial biogenesis, antioxidant enzyme expression, angiogenesis and tissue remodeling [12,13,14]. Thus, the same increase in lipid peroxidation markers may represent either an adaptive redox signal or a maladaptive response, depending on training load, recovery, nutritional status and sampling time. This distinction is particularly relevant in elite female team-sport athletes, for whom molecular evidence remains limited and often derives from small, highly selected cohorts [15]. Free radical-induced oxidation of polyunsaturated fatty acids by ROS leads to lipid peroxidation, resulting in cellular membrane damage and the formation of toxic by-products such as malondialdehyde (MDA) [16,17]. In addition, isoprostanes are generated and are considered among the most reliable biomarkers of oxidative stress in vivo. Of particular interest, due to its stability and widespread use as an indicator of oxidative damage, is 8-iso-prostaglandin F2α (8-iso-PGF2α), which is formed through the non-enzymatic peroxidation of arachidonic acid [18].

Elevated levels of 8-iso-PGF2α have been observed in numerous pathological conditions associated with oxidative stress and inflammation, including cardiovascular diseases, diabetes, neurodegenerative disorders, and autoimmune diseases. Furthermore, 8-iso-PGF2α is widely used as a marker of oxidative stress in various clinical conditions, including brain injury and inflammatory processes [19,20,21,22]. It has also been demonstrated that its levels may be modulated by dietary factors and antioxidant interventions [23,24]. In the context of physical activity, intense exercise has been shown to induce a transient increase in oxidative stress markers, including lipid peroxidation products; however, the magnitude of these changes may depend on the type of exercise and the individual’s training status [25,26].

The *ALOX* (arachidonic acid lipoxygenase) genes constitute a family of genes encoding lipoxygenase enzymes, which are non-heme iron-containing dioxygenases. These enzymes catalyze the oxidation of polyunsaturated fatty acids, such as arachidonic acid, into their corresponding hydroxy or hydroperoxy derivatives. The human genome contains six functional *ALOX* genes: *ALOX15*, *ALOX15B*, *ALOX12*, *ALOX12B*, *ALOXE3*, and *ALOX5* [27,28,29]—which have been implicated in the development of various diseases [30]. *ALOX5* is responsible for leukotriene synthesis, which plays a crucial role in the pathogenesis of inflammatory diseases, especially asthma [31]. *ALOX12* is involved in the production of lipids regulating platelet function and coagulation, and has been associated with atherosclerosis, neurodegenerative disorders, and cancer [32]. *ALOX15* contributes to the formation of anti-inflammatory mediators such as lipoxins and has been linked to diseases of the kidneys, cardiovascular system, and nervous system, and metabolic disorders [33]. A common feature of these conditions is the presence of increased oxidative stress and disturbances in lipid metabolism [30].

A better understanding of the molecular mechanisms underlying exercise-induced oxidative stress may facilitate the identification of biomarkers reflecting physiological adaptation to long-term training. In particular, biomarkers associated with lipid peroxidation and lipoxygenase pathways may complement conventional biochemical and physiological indicators by providing additional insight into redox-related adaptive processes. Although their clinical and practical utility requires further validation, such molecular markers may ultimately contribute to training load monitoring, recovery assessment, and the development of more individualized exercise strategies. Despite the growing number of studies investigating both oxidative stress markers and the expression of genes involved in arachidonic acid metabolism, there remains a limited number of studies examining these parameters simultaneously in the context of physical exercise. In particular, there is a lack of research focusing on physically active women, highlighting a significant gap in current knowledge. Therefore, the present pilot study aimed to compare plasma levels of 8-iso-PGF2α before and after a 10-week preparatory training period and to assess potential associations with *ALOX5*, *ALOX12* and *ALOX15* expression in biological material obtained from professional female volleyball players. We hypothesized that preparatory training would increase 8-iso-PGF2α and selectively modulate lipoxygenase-related transcripts, with the strongest response expected for *ALOX5* because of its role in leukotriene biosynthesis and leukocyte-mediated inflammatory signaling. We further hypothesized that these changes would show exploratory discriminatory value for differentiating the post-break and post-training states; however, these analyses were interpreted cautiously because of the paired pilot design.

## 2. Results

**Figure 1:**
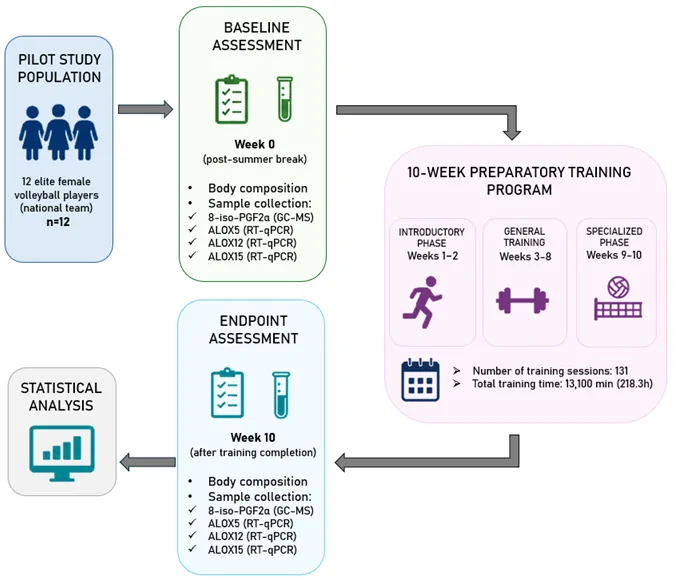
Study design and experimental workflow. Abbreviations: GC–MS, gas chromatography–mass spectrometry; RT-qPCR, reverse transcription quantitative polymerase chain reaction; 8-iso-PGF2α, 8-iso-prostaglandin F2α; *ALOX5*, arachidonate 5-lipoxygenase gene; *ALOX12*, arachidonate 12-lipoxygenase gene; *ALOX15*, arachidonate 15-lipoxygenase gene.

The overall study design is summarized in Figure 1. The flowchart illustrates participant enrollment, the 10-week preparatory training program, the timing of baseline and endpoint assessments, and the experimental workflow used in the present study.

**Figure 2:**
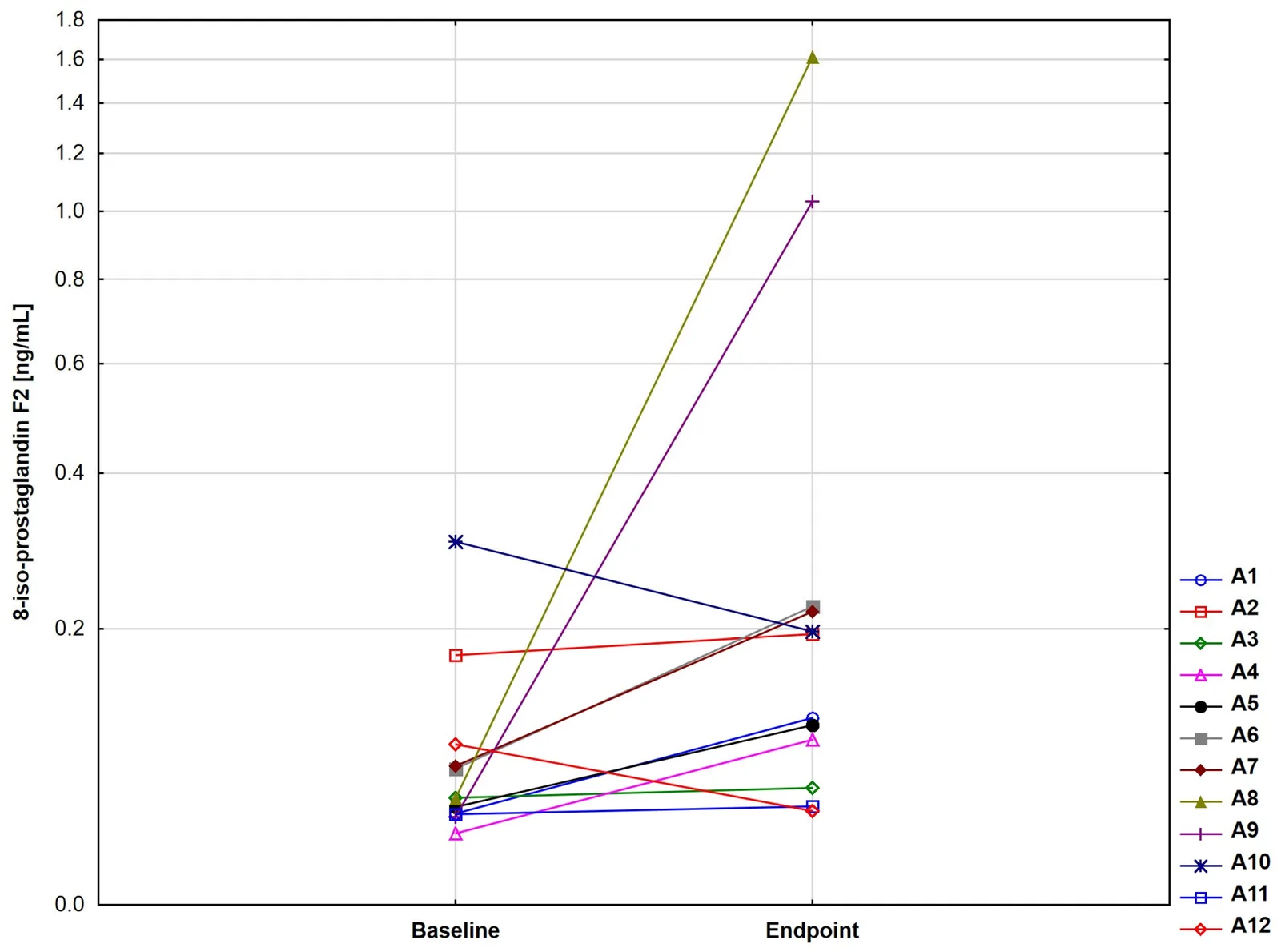
Paired plasma 8-iso-PGF2α (8-iso-prostaglandin F2α) values at baseline and endpoint. Lines connect measurements from the same athlete; labels A1–A12 identify individual athletes.

**Figure 3:**
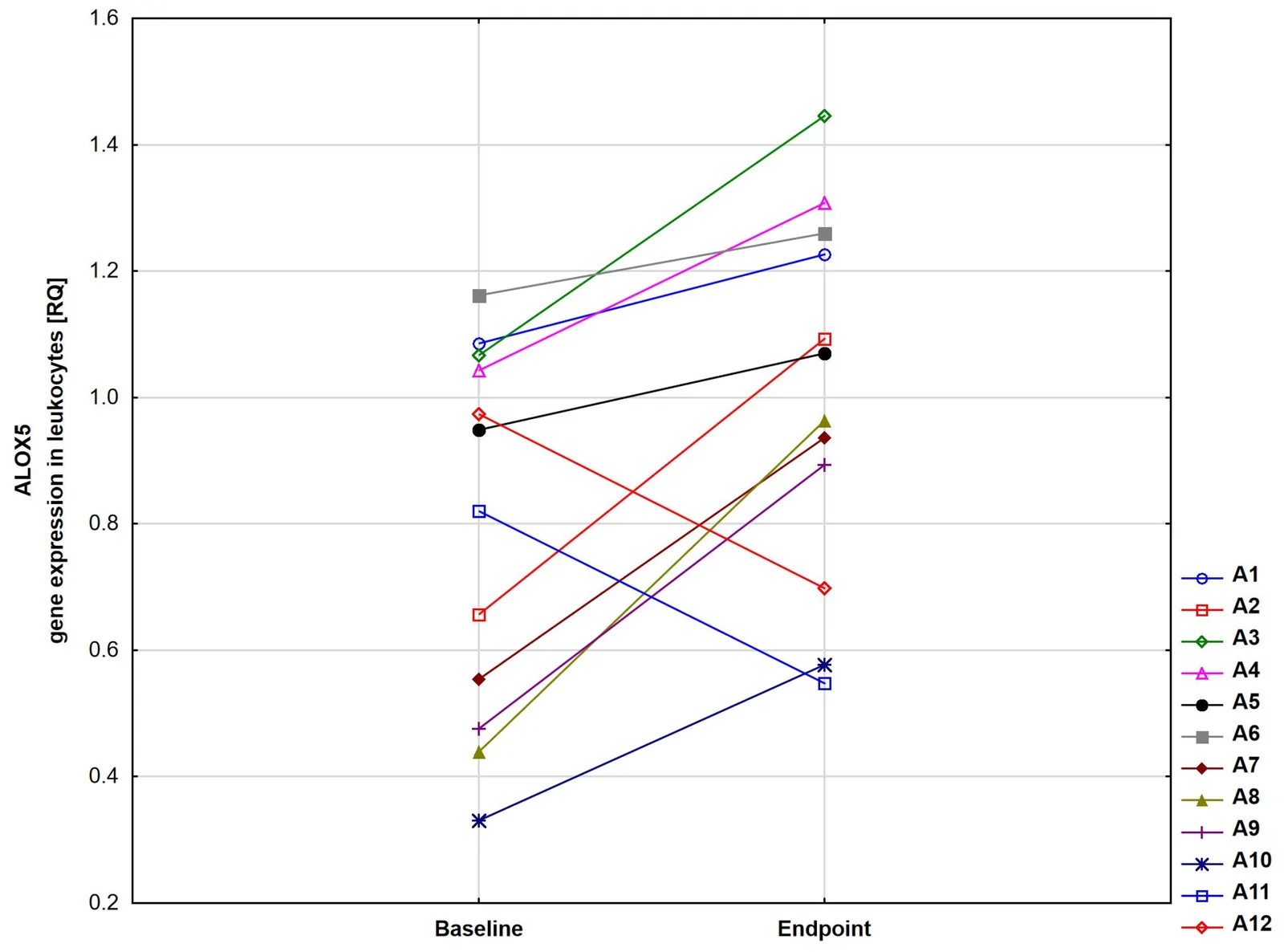
Paired leukocyte *ALOX5* (arachidonate 5-lipoxygenase gene) relative-expression values at baseline and endpoint. Lines connect measurements from the same athlete; labels A1–A12 identify individual athletes. RQ, relative quantification.

**Figure 4:**
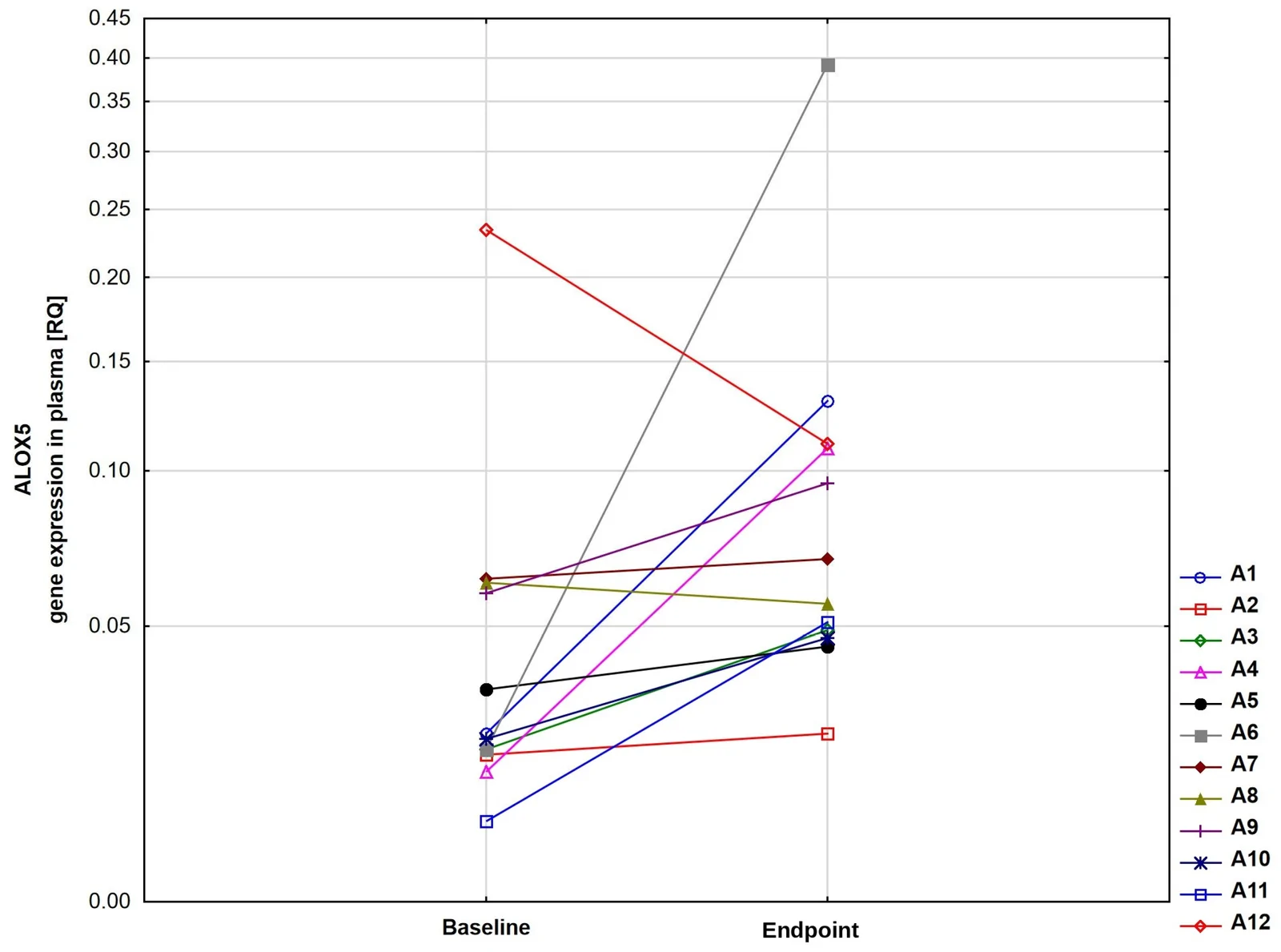
Paired plasma-derived *ALOX5* (arachidonate 5-lipoxygenase gene) relative transcript-expression values at baseline and endpoint. Lines connect measurements from the same athlete; labels A1–A12 identify individual athletes. RQ, relative quantification.

Individual paired trajectories are shown in Figure 2, Figure 3 and Figure 4. Median plasma 8-iso-PGF2α was 0.06 [0.05–0.09] ng/mL at baseline and 0.16 [0.08–0.22] ng/mL at endpoint (unadjusted *p* = 0.03). Mean leukocyte *ALOX5* expression was 0.80 ± 0.29 RQ at baseline and 1.00 ± 0.29 RQ at endpoint (*p* = 0.02), while median plasma-derived *ALOX5* expression was 0.03 [0.02–0.06] RQ and 0.06 [0.05–0.11] RQ, respectively (*p* = 0.04). These three comparisons had unadjusted *p* values below 0.05, but none met the Bonferroni-corrected threshold of 0.01 for the five-outcome family. *ALOX12* (*p* = 0.21) and *ALOX15* (*p* = 0.43) also did not meet the corrected threshold. Details are provided in Table 1.

Following the training period, plasma levels of 8-iso-PGF2α increased markedly, showing an approximate 165–170% rise relative to baseline (*p* = 0.03). *ALOX5* expression exhibited significant upregulation in whole-blood leukocytes, with an increase of approximately 25% (*p* = 0.02). In contrast, *ALOX12* median expression values in leukocytes showed a moderate decrease of 20%, which did not reach statistical significance (*p* = 0.21). Although *ALOX15* median expression values in leukocytes increased by more than 100% over the training period, this change was not statistically significant (*p* = 0.43). Given the significant increase in leukocyte *ALOX5* expression between the training periods, its presence was further examined in plasma-derived RNA. Plasma-derived *ALOX5* transcript levels approximately doubled (*p* = 0.04) at the end of the training period. Details are presented in Table 1 and Figure 2, Figure 3 and Figure 4.

There was no significant correlation between leukocyte *ALOX5* expression and plasma-derived *ALOX5* transcript expression (Spearman’s rank correlation coefficient (rs) = 0.17). At baseline, there were no significant correlations between plasma levels of 8-iso-PGF2α and leukocyte expression of *ALOX5*, *ALOX12* or *ALOX15*, nor with plasma-derived *ALOX5* transcript expression. At endpoint, no significant correlations were observed between the studied molecules. Details are presented in Table 2. Correlations between body composition parameters and the studied molecules were evaluated; however, no significant associations were identified for the remaining body composition parameters or aerobic capacity. Details are presented in Table 3.

**Table 2:** Spearman’s rank correlations between the studied molecules.

Training Period	*ALOX5* [RQ in Leukocytes]	*ALOX12* [RQ in Leukocytes]	*ALOX15* [RQ in Leukocytes]	*ALOX5* [RQ in Plasma]
Baseline 8-iso-PGF2α	−0.24	−0.08	0.12	0.25
Endpoint 8-iso-PGF2α	0	−0.5	0.08	0.23

Abbreviations: 8-iso-PGF2α, 8-iso-prostaglandin F2α; *ALOX*, arachidonic acid lipoxygenases gene; RQ, relative quantification.

**Table 3:** Spearman’s rank correlations between levels of studied molecules and demographic and training parameters.

**Baseline**							
	**Weight**	**FAT%**	**Fat Mass**	**FFM**	**TBW**	**BMR**	**VO_2_max**
8-iso-PGF2α	0.25	0.55	0.38	0.3	0.3	0.22	−0.57
*ALOX5* [RQ in leukocytes]	−0.29	−0.25	−0.26	−0.22	−0.22	−0.13	0.2
*ALOX12* [RQ in leukocytes]	0.48	0.38	0.46	0.37	0.37	0.45	−0.16
*ALOX15* [RQ in leukocytes]	−0.24	−0.07	−0.24	−0.12	−0.12	−0.08	0.09
*ALOX5* [RQ in plasma]	0.25	0.55	0.38	0.3	0.30	0.42	−0.57
**Endpoint**							
	**Weight**	**FAT%**	**Fat Mass**	**FFM**	**TBW**	**BMR**	**VO_2_max**
8-iso-PGF2α	0.08	0.33	0.26	−0.02	−0.08	−0.09	0.03
*ALOX5* [RQ in leukocytes]	−0.01	−0.28	−0.2	0.12	0.15	0.15	−0.01
*ALOX12* [RQ in leukocytes]	0.17	−0.13	−0.01	0.13	0.17	0.2	0.1
*ALOX15* [RQ in leukocytes]	−0.26	0.06	0.1	−0.29	−0.27	−0.22	−0.34
*ALOX5* [RQ in plasma]	0.04	−0.11	−0.08	0.12	0.14	0.05	0.16

Data are presented as Spearman’s correlation coefficients. Abbreviations: 8-iso-PGF2α, 8-iso-prostaglandin F2α; *ALOX*, arachidonic acid lipoxygenases gene; RQ, relative quantification; FAT%, body fat percentage; FFM, fat-free mass; TBW, total body water; BMR, basal metabolic rate; VO_2_max, maximal oxygen uptake.

## 3. Discussion

This exploratory pilot study provides novel evidence that a 10-week preparatory training period in professional female volleyball players is associated with enhanced lipid peroxidation, as demonstrated by elevated plasma levels of 8-iso-PGF2α, alongside a selective upregulation of *ALOX5* expression in both whole-blood leukocytes and plasma-derived RNA. These findings highlight a potentially important role of *ALOX5* in exercise-induced oxidative processes. Notably, no significant training-related alterations were observed for *ALOX12* or *ALOX15*, underscoring the specificity of the *ALOX5* response.

Collectively, these results indicate that preparatory training may promote lipid-oxidative remodeling linked specifically to the 5-lipoxygenase pathway, rather than inducing a broad activation of all lipoxygenase-related transcripts. The observed rise in 8-iso-PGF2α should be considered in the context of exercise-induced redox signaling rather than interpreted solely as evidence of oxidative damage. F2-isoprostanes are well-established products of arachidonic acid peroxidation; however, their responses after exercise are influenced by the timing of sample collection, the biological matrix analyzed, the analytical method used, and whether acute or chronic training adaptations are being evaluated [26,34]. In elite athletes, an increase after training may reflect greater lipid oxidative turnover associated with adaptation, particularly when considered alongside improvements in body composition and aerobic capacity. This interpretation aligns with the concept that redox signals generated during muscle contraction can contribute to mitochondrial adaptations, antioxidant defense, and tissue remodeling, whereas excessive oxidative stress or inadequate recovery may have maladaptive consequences [12,13,14].

Mechanistically, the increase in 8-iso-PGF2α may point to intensified arachidonic acid turnover at the intersection of non-enzymatic lipid peroxidation and enzymatically regulated oxylipin signaling. This distinction is important because oxidized lipid derivatives may act not only as indicators of molecular damage but also as context-dependent signals involved in mitochondrial remodeling, activation of antioxidant systems, and communication between immune cells during repeated training exposure [12,13,14,35,36]. In the present group of athletes, the simultaneous improvement in body composition and aerobic fitness supports the interpretation that the endpoint profile reflects training-related lipid-oxidative remodeling rather than isolated oxidative injury. Nevertheless, this conclusion remains inferential because antioxidant enzymes, cytokines, recovery-related markers, and downstream oxylipins were not measured in parallel.

The selective upregulation of *ALOX5* is biologically credible, as 5-lipoxygenase is mainly expressed in leukocytes and catalyzes the first steps of leukotriene formation from arachidonic acid [30,31]. In a training context, this response should not necessarily be viewed as pathological. Instead, it may reflect transient activation of lipid-mediator pathways involved in leukocyte recruitment, tissue repair, and redox-sensitive remodeling. The lack of corresponding changes in *ALOX12* and *ALOX15* suggests that the post-training response was not a generalized activation of lipoxygenase genes, but rather a more selective modulation of the 5-lipoxygenase axis. This specificity reinforces the interpretation of *ALOX5* may be involved in molecular responses associated with training adaptation, while also emphasizing the need for future studies to quantify downstream lipid mediators, including leukotriene B4, 5-HETE, 12/15-HETE, lipoxins, and resolvins. Importantly, the 5-lipoxygenase pathway should be understood as part of a dynamic inflammatory and resolution-related network rather than as an exclusively pro-inflammatory cascade. Although 5-lipoxygenase drives leukotriene production, including LTB4 and cysteinyl leukotrienes, lipoxygenase-dependent metabolism may also interact with pro-resolving pathways, including lipoxin and resolvin formation, depending on substrate availability, cellular environment, and transcellular biosynthesis [33,35,37,38]. This is particularly relevant to exercise physiology, as muscle loading and repair depend on coordinated leukocyte recruitment, local inflammatory signaling, and subsequent resolution rather than complete suppression of inflammation [36,37,38].

Although both plasma 8-iso-PGF2α concentrations and *ALOX5* expression increased following the training period, no significant correlation was observed between these variables. This finding suggests that these biomarkers may reflect complementary rather than directly coupled aspects of the physiological response to long-term exercise. Whereas 8-iso-PGF2α is considered a marker of non-enzymatic lipid peroxidation and oxidative stress, *ALOX5* expression reflects activation of the enzymatic lipoxygenase pathway involved in inflammatory and immunoregulatory signaling. Therefore, both measures may respond to the same training stimulus through partially independent biological mechanisms rather than through a direct causal relationship. Given the pilot nature of the present study, this interpretation should be considered exploratory and requires confirmation in larger longitudinal studies integrating transcriptomic, lipidomic, and functional analyses.

Findings from targeted lipidomic studies further support this interpretation. Resistance exercise has been reported to modify circulating inflammatory and pro-resolving lipid mediators, while exposure to non-steroidal anti-inflammatory drugs may alter this mediator profile [39]. In trained football players, training status, acute exercise, and docosahexaenoic acid intake influenced both plasma eicosanoid concentrations and eicosanoid production by peripheral blood mononuclear cells [40]. Similarly, in a randomized exercise-training setting, acute and long-term exercise produced distinct plasma oxylipin and endocannabinoid profiles: acute exercise increased several classes of lipid mediators, whereas longer-term training reduced fasting omega-6-derived oxylipins in young sedentary adults [41]. These observations highlight the importance of sample timing in relation to the most recent exercise bout when interpreting changes in *ALOX5* expression and 8-iso-PGF2α levels.

The absence of significant changes in *ALOX12* and *ALOX15* is also informative. It indicates that the molecular pattern observed after training is unlikely to represent nonspecific activation of all lipoxygenase transcripts but instead suggests a more focused response involving the 5-lipoxygenase pathway. However, *ALOX12* and *ALOX15* expression and activity may be highly dependent on cell type and biological context, and their lipid products may become more evident during stages of tissue repair or inflammation resolution. Future studies should therefore combine transcript-level measurements with targeted quantification of LTB4, cysteinyl leukotrienes, 5-HETE, 12-HETE, 15-HETE, lipoxin A4, resolvin D1/D2, and related specialized pro-resolving mediators.

The plasma-derived *ALOX5* transcript signal should be interpreted with particular caution. Circulating RNA may originate from several sources, including leukocytes, platelets, extracellular vesicles, or other cell-free RNA fractions. Moreover, RT-qPCR cannot establish the cellular origin of the transcript, nor does it provide information on 5-lipoxygenase protein abundance or enzymatic activity. Therefore, plasma-derived *ALOX5* expression should be regarded as a minimally invasive candidate molecular readout that may complement leukocyte-based measurements, rather than as a direct indicator of 5-lipoxygenase activity.

From the perspective of future biomarker research, the present findings should be regarded as preliminary and hypothesis-generating, suggesting that changes in 8-iso-PGF2α and *ALOX5* may reflect physiological responses associated with long-term training and require independent validation. Reliable implementation of molecular measures in elite sport requires analytical robustness, biological plausibility, individualized reference ranges, standardized sampling conditions, predefined decision thresholds, and validation in independent cohorts [42,43,44]. The current results provide biological plausibility and include a validated analytical method for 8-iso-PGF2α, but they do not yet define practical monitoring thresholds for training or clinical decision-making. A longitudinal panel combining 8-iso-PGF2α, *ALOX5*, conventional inflammatory markers, training-load metrics, recovery indices, and performance outcomes would likely offer greater interpretive value than any single molecular measure alone.

Previous research in elite female volleyball players has shown that training periods can alter oxidative stress-related biomarkers, supporting the relevance of redox monitoring in this athletic population [15]. However, most studies to date have focused primarily on systemic biochemical markers rather than lipoxygenase-related gene expression. The present study expands this area by combining GC–MS-based measurement of a lipid peroxidation marker with analysis of *ALOX* transcript responses in leukocytes and plasma-derived RNA. Therefore, despite the pilot nature of the study and the limited sample size, these findings provide a molecular foundation for future longitudinal research integrating redox biomarkers, eicosanoid profiling, transcriptomics, and performance-related outcomes.

The female-athlete context is especially important. Nutritional status, energy availability, iron status, oral contraceptive use, menstrual-cycle characteristics, and recovery may all influence inflammatory, redox, and hematological biomarkers [45,46,47,48]. Recent studies in women suggest that oral contraceptive use and exercise can affect oxidative-stress biomarkers, while data from highly trained female athletes indicate that some inflammatory and hematological markers may remain relatively stable across menstrual-cycle phases but may be modified by combined oral contraceptive use [46,47,48]. For this reason, future studies involving professional female volleyball players should prospectively document menstrual-cycle phase, hormonal contraception, menstrual symptoms, dietary antioxidant intake, omega-3 fatty-acid intake, medication use, recent match exposure, and sleep or recovery status. A logical next step would be to conduct a season-long repeated-measures study with blood sampling performed after standardized recovery periods, together with concurrent monitoring of external and internal training load and targeted lipidomic profiling. Such an approach would help distinguish acute post-exercise responses from chronic training adaptations and would enable the development of individualized reference intervals. It would also help determine whether *ALOX5* upregulation reflects beneficial adaptation, insufficient recovery, or a normal immunometabolic response to preparatory training load.

The present study has several limitations. The small cohort and absence of a non-training control group reduce the precision, statistical power, and generalizability of the findings. Recruitment in elite sport is inherently constrained because the eligible population may be limited to a single professional team or national squad, and repeated biological sampling must be coordinated with training and competition schedules without disrupting performance or recovery. These constraints, including the recognized “small-sample–small-relevant-effect” problem, are well documented in elite-sport research [49,50,51], and comparable longitudinal biomarker studies have included approximately 12–16 athletes [15,52,53]. Nevertheless, such precedent does not establish the statistical adequacy of the present sample or eliminate the risk of imprecise and unstable estimates; it merely illustrates a recurrent practical recruitment ceiling. Due to the exploratory nature of the study and the limited sample size, nominal *p*-values are reported. Accordingly, the findings should be regarded as preliminary and hypothesis-generating and require validation in larger, prospectively designed controlled cohorts.

The intermediate sampling points were not included, making it impossible to distinguish cumulative training adaptations from seasonal, nutritional, or recovery-related influences. Menstrual-cycle phase, hormonal contraceptive use, diet, antioxidant supplementation, and recent training or match load were also not controlled. Furthermore, classical oxidative-stress markers, such as SOD, MDA, and TOC, as well as downstream eicosanoids and protein expression, were not assessed. Future studies should incorporate repeated sampling, appropriate control groups, broader molecular profiling, and performance outcomes to clarify exercise-induced lipid peroxidation and lipoxygenase-related pathways.

## 4. Materials and Methods

### 4.1. Study Design

The present pilot study is based on our previous research conducted in the same cohort of elite female volleyball players using the same biological samples collected during a 10-week preparatory training period. The sample size was consistent with previous longitudinal studies conducted in elite athletes [52,53,54]. This reflects the limited availability of athletes competing at the highest level, for whom relatively small cohorts are typical in longitudinal research. While our previous studies focused on different molecular biomarkers, including circulating microRNAs, *IGF1R* gene expression, and long non-coding RNAs, the present study investigates, for the first time, lipid peroxidation (8-iso-PGF2α) and the expression of the lipoxygenase-related genes *ALOX5*, *ALOX12*, and *ALOX15*. Thus, the present work addresses a distinct biological question concerning oxidative lipid metabolism and molecular adaptations to long-term training. The study was approved by the Bioethics Committee of the University of Rzeszów (Protocol No. 3/11/2017).

The pilot study was conducted in a cohort of 12 elite professional female volleyball players competing at the highest national and international levels and representing the national team. All team members were enrolled during a preseason preparatory period following the summer break, ensuring population homogeneity. Participants had a mean training experience of 13.7 ± 6.4 years. Baseline demographic and anthropometric characteristics have been previously reported [55,56]. All athletes were injury-free and reported no pharmacological treatment. Written informed consent was obtained from all participants. Owing to the restricted availability of world-class athletes and stringent inclusion criteria, the sample size was limited, consistent with similar elite-sport studies.

The intervention comprised a 10-week structured training program including a 2-week introductory phase, a 6-week general preparatory phase, and a 2-week specialized phase. Measurements were performed at two time points: baseline (immediately post-break) and after completion of the preparatory period, allowing assessment of training-induced biomarker changes under controlled physiological conditions.

Across 10 weekly microcycles, athletes completed 131 training sessions totaling 13,100 min (218.3 h), with an additional 1320 min dedicated to recovery. Training load distribution progressed from predominantly aerobic work (weeks 1–3) to balanced aerobic-resistance training (weeks 4–6), and finally to power-focused training (weeks 7–10). Physiological and biochemical monitoring included VO_2_max estimation using a standardized incremental field test and venous blood analysis for creatine kinase and cortisol at both time points. Body composition was assessed using bioelectrical impedance analysis with a Tanita BC-418 MA analyzer (Tanita Corporation, Tokyo, Japan). Training methodology, including the training schedule, intensity distribution, and training intensity intervals applied throughout the 10-week preparatory period, as well as the assessment procedures, body composition, and aerobic capacity results for this cohort, has been reported briefly in Table 4 and in detail previously [55,56].

To reduce pre-analytical variability, blood sampling was performed according to the standardized protocol used in the previously described cohort [55,56]. Sampling was conducted at the two predefined time points, baseline and endpoint, using the same biological matrix and analytical workflow.

### 4.2. Materials

All chemicals and reagents for GC/MS analysis were of analytical grade. 15(S)-8-iso-PGF_2_α, [3,3′,4,4′-^2^H_4_]-15(S)-8-iso-PGF_2_α, and the 8-isoprostane affinity column with column buffer, storage buffer, and eluent were purchased from Cayman Chemical (Ann Arbor, MI, USA). Potassium hydroxide, potassium dihydrogen phosphate, and ethyl acetate were obtained from Chempur (Piekary Śląskie, Poland). Anhydrous ethanol, anhydrous acetonitrile, and anhydrous methanol were supplied by Honeywell (Seelze, Germany). Hydrochloric acid, N,N-diisopropylethylamine, pentafluorobenzyl bromide (PFB-Br), and N,O-bis(trimethylsilyl)trifluoroacetamide (BSTFA) were purchased from Sigma-Aldrich (St. Louis, MO, USA). Ultrapure water (resistivity > 18.2 MΩ·cm) was obtained from a water purification system (Hydrolab Sp. z o.o., Straszyn, Poland). GC–MS analyses were performed using a GC-MS-QP2010 Ultra gas chromatograph coupled with a mass spectrometer (Shimadzu, Kyoto, Japan) equipped with a Zebron ZB-5MS capillary column (30 m × 0.25 mm × 0.25 µm; Phenomenex, Torrance, CA, USA).

The following reagents were used for RNA isolation, purification, reverse transcription, and qPCR analysis: RNA Extracol (EURx, Gdańsk, Poland), chloroform and isopropanol (Chempur, Piekary Śląskie, Poland), miRNeasy Serum/Plasma Advanced Kit (Qiagen, Hilden, Germany), PrimeScript RT Reagent Kit (Takara Bio Inc., Kusatsu, Shiga, Japan), and Fast SG qPCR Master Mix (EURx, Gdańsk, Poland). Molecular biology-grade water was used for RNA resuspension. Quantitative PCR analyses were performed using the QuantStudio 5 Real-Time PCR System (Thermo Fisher Scientific, Waltham, MA, USA).

### 4.3. Isoprostane Quantification by GC–MS

The determination of 15(S)-8-iso-prostaglandin F_2_α was carried out using a validated extraction and derivatization procedure adapted from a previously published GC–MS method [57]. A standard solution of 15(S)-8-iso-PGF_2_α (50 ng/mL) and the deuterated internal standard [3,3′,4,4′-^2^H_4_]-15(S)-8-iso-PGF_2_α (50 ng/mL) were added to each sample in a volume of 20 µL.

Frozen plasma samples were thawed on ice and centrifuged at 4 °C (4500× *g*, 5 min) to remove particulate material. After gentle mixing, 0.5 mL of plasma was transferred into 12 mL polypropylene tubes and spiked with the internal standard. The samples were kept on ice for 30 min to allow equilibration. Alkaline hydrolysis of esterified isoprostanes was then performed by adding 500 µL of 3.5 M KOH, followed by incubation in a water bath at 38 °C for 1 h.

To eliminate neutral lipids, ethanol (500 µL) and chilled heptane (1 mL, 4 °C) were added to each tube. After vortex mixing for 2 min, the samples were stored at −20 °C for 30 min and then centrifuged (4500× *g*, 5 min, 4 °C). The upper heptane layer together with the lipid ring was carefully removed. This washing step was repeated once more. The remaining aqueous phase was acidified to approximately pH 3 using 300 µL of 5 M HCl and 600 µL of 1 M KH_2_PO_4_.

Extraction of analytes was performed twice with 3 mL portions of ethyl acetate. Each extraction was followed by vortex mixing for 2 min and centrifugation (4500× *g*, 5 min, 4 °C). The organic layers were pooled and evaporated to dryness.

The dry residue was dissolved in 50 µL of ethanol, followed by the addition of 500 µL of immunoaffinity column (IAC) buffer and 500 µL of dichloromethane. After mixing and centrifugation, the upper aqueous phase was collected. A second extraction with 800 µL of IAC buffer was performed and the supernatants were combined. The resulting solution was applied to immunoaffinity columns previously washed with column buffer and distilled water. After loading, the columns were rinsed and the analytes were eluted with 4 mL of elution solvent (2 × 2 mL). The eluates were concentrated under a gentle nitrogen stream at 45 °C. If necessary, samples were stored at −20 °C prior to final evaporation to dryness and subsequent derivatization.

Derivatization was carried out in two consecutive steps. First, pentafluorobenzyl ester formation was performed by dissolving the dry extract in 100 µL of anhydrous acetonitrile and adding 10 µL of anhydrous methanol, followed by 10 µL of N,N-diisopropylethylamine and 10 µL of pentafluorobenzyl bromide (PFB-Br, 0.5 mg/mL in acetonitrile). The reaction mixture was incubated at 30 °C for 60 min in sealed glass vials. After evaporation of solvents under nitrogen, trimethylsilyl ether formation was performed by adding 50 µL of BSTFA and incubating the samples at 60 °C for another 60 min. The final PFB-TMS derivatives were transferred into GC vials and analyzed by GC–MS. The GC–MS analytical parameters used for the determination of 15(S)-8-iso-prostaglandin F_2_α are presented in Table 5. The GC oven temperature program started at 180 °C with an initial hold of 1.5 min, followed by a ramp of 10 °C/min to 340 °C, where it was held for 10 min.

The GC-MS method was monitored using the deuterated internal standard added before extraction. The analytical validation parameters were as follows: detection limit, 0.002 ng/mL; quantitation limit, 0.007 ng/mL; calibration curve, Y = 63.73323X + 1.069761 × 10^−3^; R^2^ = 0.999; and R = 0.999. Precision was acceptable, with an intra-assay relative standard deviation (RSD) of 5.56% (N = 6) and an intra-day RSD of 6.87% (N = 6). These parameters are summarized in Supplementary Table S2.

### 4.4. Gene Expression Analysis

The selection of the *ALOX5*, *ALOX12* and *ALOX15* genes was based on their potential involvement in pathways related to arachidonic acid metabolism, isoprostane formation and lipid oxidation. Whole blood was collected and analyzed at two time points: week 1 (baseline) and week 10 (endpoint). Total RNA from blood white blood cells and plasma samples was extracted as previously described [55,56]. Briefly, 1 mL of RNA Extracol was added to the leukocyte pellet after red blood cell lysis. Total RNA was extracted using the chloroform–isopropanol method and resuspended in molecular biology-grade water. Plasma total RNA was extracted and purified using the miRNeasy Serum/Plasma Advanced Kit according to the manufacturer’s instructions. Reverse transcription was performed using the PrimeScript RT Reagent Kit in a total reaction volume of 20 µL according to the manufacturer’s instructions. Following reverse transcription, cDNA obtained from leukocytes was diluted five-fold, whereas cDNA derived from plasma samples was diluted two-fold. Target transcript expression was quantified relative to the reference gene *B2M*. Primer sequences are provided in Supplementary Table S1. Relative gene expression was calculated using the 2^−ΔΔCq^ method with respect to a calibrator sample included in each PCR run. qPCR reporting was structured according to MIQE recommendations [58,59]. Primer specificity was verified by agarose gel electrophoresis and melt-curve analysis. qPCR reactions were performed using Fast SG qPCR Master Mix. The thermal cycling conditions included an initial denaturation step at 95 °C for 3 min, followed by 40 or 45 amplification cycles depending on whether the cDNA originated from leukocytes or plasma, consisting of denaturation at 95 °C for 5 s and primer annealing/extension at 60 °C for 30 s. Melt-curve analysis was conducted after each amplification run to confirm product specificity.

### 4.5. Statistics

The distribution of quantitative variables was assessed using the Shapiro–Wilk test. Normally distributed data are presented as means ± standard deviation (SD), whereas non-normally distributed variables are expressed as medians with interquartile ranges (IQRs; 25th–75th percentile). Differences between the two training periods were analyzed using the paired-samples *t*-test or Wilcoxon signed-rank test, depending on data distribution. Correlations between continuous variables were assessed using Spearman’s rank correlation coefficient and interpreted as exploratory because of the small sample size. Statistical analyses were performed using STATISTICA software, version 13.3 (TIBCO Software Inc., Palo Alto, CA, USA). A *p*-value of less than 0.05 was considered statistically significant.

## 5. Conclusions

In conclusion, this exploratory pilot study observed nominal pre-to-post increases in plasma 8-iso-PGF2α concentrations and *ALOX5* transcript levels in leukocytes and plasma-derived RNA after a 10-week preparatory training period in elite female volleyball players, whereas no statistically detectable changes were observed in *ALOX12* or *ALOX15* expression. These findings are consistent with changes in lipid oxidative processes involving the 5-lipoxygenase pathway during long-term training. To our knowledge, this is the first pilot longitudinal study to simultaneously evaluate plasma 8-iso-PGF2α concentrations together with *ALOX5*, *ALOX12*, and *ALOX15* expression in elite female volleyball players during a preparatory training period. The observed increase in 8-iso-PGF2α is consistent with previous evidence indicating that intensive exercise is associated with enhanced lipid peroxidation, while the selective upregulation of *ALOX5* is consistent with its potential involvement in exercise-induced inflammatory and oxidative adaptations. Plasma 8-iso-PGF2α concentrations and *ALOX5* transcript levels in plasma-derived RNA may reflect physiological responses associated with the preparatory training period; however, their biological significance and potential utility for monitoring training adaptation require further investigation. These hypothesis-generating findings require confirmation in larger, prospectively designed controlled studies incorporating repeated sampling, measures of training load and performance, female-specific physiological factors, and downstream eicosanoid and protein analyses before their robustness, biological significance, and potential utility for monitoring training adaptation can be established.

## Figures and Tables

**Table 1:** Paired baseline-to-endpoint comparisons for plasma 8-iso-PGF2α and selected transcript-expression measures.

Parameter	Baseline	Endpoint	Test	Unadjusted *p*
8-iso-PGF2α [ng/mL]	0.06 [0.05–0.09]	0.16 [0.08–0.22]	Wilcoxon signed-rank	0.03
*ALOX5* in leukocytes [RQ]	0.80 ± 0.29	1.00 ± 0.29	Paired *t*-test	0.02
*ALOX5* in plasma-derived RNA [RQ]	0.03 [0.02–0.06]	0.06 [0.05–0.11]	Wilcoxon signed-rank	0.04
*ALOX12* in leukocytes [RQ]	2.45 [1.60–6.01]	1.96 [1.49–4.22]	Wilcoxon signed-rank	0.21
*ALOX15* in leukocytes [RQ]	0.15 [0.04–1.04]	0.36 [0.20–0.91]	Wilcoxon signed-rank	0.43

Data are presented as mean ± SD or median [interquartile range]. Because five paired biomarker comparisons were evaluated without a prespecified primary endpoint, family-wise α = 0.05 was controlled using a Bonferroni threshold of 0.01. No comparison met the corrected threshold. Unadjusted two-sided *p* values are shown for transparency. Abbreviations: 8-iso-PGF2α, 8-iso-prostaglandin F2α; *ALOX*, arachidonic acid lipoxygenase gene; RQ, relative quantification.

**Table 4:** Condensed description of the 10-week preparatory training exposure.

Weeks/Phase	Coach-Planned Content Distribution	Recorded Exposure/Phase Note
Weeks 1–3 Introductory/early general preparation	80% aerobic; 20% strength resistance	Includes the 2-week introductory phase and transition to general preparation.
Weeks 4–6 General preparation	60% aerobic; 40% resistance	Peak-volume microcycle: week 4, 1800 min across 12 sessions.
Weeks 7–10 Late general/specialized preparation	80% power; 20% aerobic	Lowest-volume microcycle: week 8, 780 min across 12 sessions.
Overall	Progression from aerobic and strength-resistance work toward power-focused preparation	131 sessions; 13,100 min (218.3 h) of training; 1320 min allocated to recovery methods.

**Table 5:** GC–MS system parameters used for the determination of 15(S)-8-iso-prostaglandin F_2_α.

Injection mode	Splitless
Carrier gas	Helium
Linear velocity	40.4 mL/min
Injection volume	1 µL
Injection temperature	280 °C
Ion source temperature	190 °C
Interface temperature	220 °C
Detector voltage	0.3 kV
Operating mode	SIM
Identified ions	573.4 *m*/*z*; 569.4 *m*/*z*

## Data Availability

The original contributions presented in this study are included in the article/Supplementary Materials. Further inquiries can be directed to the corresponding author.

## Supplementary Materials

The following supporting information can be downloaded at https://www.mdpi.com/article/10.3390/ijms27167104/s1.
