# Supplementary material for: 8-Iso-Prostaglandin F2α and Lipoxygenase Gene Expression as Candidate Molecular Markers of Training Adaptation in Professional Volleyball Players: A Pilot Study
Source: Int J Mol Sci. 2026 Aug 8;27(16):7104. doi: 10.3390/ijms27167104 (PMC13513359; doi:10.3390/ijms27167104)
Supplement: Supplementary file 1 [file ijms-27-07104-s001.zip › ijms-4372542-supplementary.pdf]

Supplementary data

Table S1. *Primer sequences used for RT-qPCR assays.*

Target name	Forward primer sequence (5'→3')	Reverse primer sequence (5'→3')
**B2M**	AGATGAGTATGCCTGCCGTG	TCATCCAATCCAAATGCGGC
**ALOX5**	GGCACTGACGACTACATCTACC	TATGAATCCACCGCGCCAC
**ALOX12**	GGCTGGACTTTGAATGGACAC	ACTGGCGAACCTTCTCAGC
**ALOX15**	ATTCCCCACCTGCGATACAC	CCAGTGCTCATTATCTGGTCG

Table S2. *GC-MS method validation parameters for 15(S)-8-iso-prostaglandin F2α quantification.*

**Detection limit (LOD)**	0.002 ng/mL
**Quantification limit (LOQ)**	0.007 ng/mL
**Calibration equation**	Y = 63.73323X + 1.069761 x10^{-3}
**Coefficient of determination (R^2)**	0.999
**Correlation coefficient (r)**	0.999
**Repeatability (intra-day precision) (N=6)**	5.56%
**Intermediate precision (inter-day precision) (N=6)**	6.87%
